# The quality and reliability of short videos about passive smoking on Bilibili and TikTok: A cross-sectional study

**DOI:** 10.18332/tid/225461

**Published:** 2026-08-05

**Authors:** Weichu Zhang, Danting Zheng, Jun Zhang

**Affiliations:** 1 Nursing Department, Sir Run Run Shaw Hospital, Zhejiang University School of Medicine, Hangzhou, China

**Keywords:** Bilibili, cross-sectional, passive smoking, secondhand smoking, TikTok (Douyin)

## Abstract

**INTRODUCTION:**

Passive smoking is associated with substantial morbidity and mortality, yet public awareness of its health risks remains limited. As social media increasingly serves as a source of health information, this study evaluated the quality and reliability of passive smoking-related short videos on two major Chinese platforms, Bilibili and Douyin (TikTok in China).

**METHODS:**

In this cross-sectional study, the top 100 videos for each Chinese search term (‘passive smoking’ and ‘secondhand smoking’) were retrieved from Bilibili and Douyin on 10 June 2026. After excluding duplicates, irrelevant videos, promotional videos, and videos shorter than 30 seconds, eligible videos were analyzed for basic characteristics and engagement metrics. Two independent reviewers assessed video quality using the Global Quality Scale (GQS), modified DISCERN (mDISCERN), Journal of the American Medical Association (JAMA) benchmark criteria, and Video Information and Quality Index (VIQI). Comparisons were performed using non-parametric tests, and correlations were examined using Spearman analyses.

**RESULTS:**

A total of 157 videos were included (59 Bilibili, 98 Douyin). Bilibili videos were significantly longer than Douyin videos (median: 130.0 vs 73.0 s, Z= -3.93, p<0.001), whereas Douyin videos received more comments (median: 89.0 vs 27.0, Z= -2.48, p<0.05) and shares (median: 1829.5 vs 72.0, Z= -4.88, p<0.001). Overall, video quality and reliability were moderate. No significant differences were observed between platforms in GQS, mDISCERN, or JAMA. However, Douyin videos achieved significantly higher VIQI scores than Bilibili videos (median: 12.0 vs 11.0, Z= -3.37, p<0.001). Videos produced by science communicators and doctors consistently demonstrated higher quality than those uploaded by lay users. Engagement metrics were not associated with video quality or reliability.

**CONCLUSIONS:**

Passive smoking-related videos on Douyin and Bilibili provide moderate-quality health information. Videos from science communicators and doctors were of higher quality than those from lay users. Video popularity does not reflect information reliability.

## INTRODUCTION

Passive smoking, also known as secondhand smoke exposure, refers to the involuntary inhalation of smoke emitted from burning tobacco products and exhaled by smokers^1-3^. Despite substantial global tobacco control efforts, passive smoking remains a major public health concern, affecting both adults and children worldwide^3,4^. Exposure to secondhand smoke has been associated with numerous adverse health outcomes, including cardiovascular disease, chronic respiratory conditions, lung cancer, and perinatal morbidities^4-7^. Vulnerable populations such as pregnant women, infants, and older adults are particularly susceptible to its harmful effects^4,6^. The World Health Organization estimates that millions of non-smokers are regularly exposed to secondhand smoke, resulting in a considerable burden of preventable morbidity and mortality^1,8^. Given its widespread prevalence and well-established health consequences, improving public awareness and understanding of passive smoking remains a critical component of tobacco control strategies.

The rapid expansion of social media has transformed the way individuals’ access to health information^9^. Social media platforms have become particularly influential due to their ability to deliver information in an engaging and easily accessible format^10,11^. Globally, YouTube remains the largest video-sharing platform and serves as an important source of health-related information for the public^10^. Furthermore, China possesses the world’s largest internet population and maintains one of the most active social media user bases globally, making online platforms a primary source of health information for the public. In China, however, the social media ecosystem differs from the outside^12^. As YouTube is unavailable in China, Bilibili is China’s equivalent to YouTube. It started from a niche video-sharing community into one of China’s leading online video platforms, particularly among younger and well-educated audiences^9^.

Douyin, another fast-growing social media platform, is TikTok’s operation in China. It is now one of the most popular short-video platforms, attracting hundreds of millions of active users every day^9^. Both platforms host a vast amount of medical and health-related content. Unlike traditional medical education dissemination, no peer-review and supervision may give rise to inaccurate, incomplete, or misleading content, thereby contributing to misconceptions, inappropriate health behaviors, or poor decision-making^10^. Therefore, systematic evaluations of health-related videos on popular social media platforms are needed.

This study aimed to explore the content, quality, and reliability of short videos related to passive smoking on two popular Chinese social media platforms, Bilibili and Douyin. The study is set in Chinese context because smoking in public is still very common in China, despite tobacco control measures being strengthened in recent years^13^.

## METHODS

### Study design and data collection

This cross-sectional study was designed and reported following the STROBE Statement^14^. The checklist can be seen in Supplementary file Table 1. The Chinese translations for ‘passive smoking’ and ‘secondhand smoking’ were used as the search terms on two Chinese social media platforms: Douyin (Chinese version of TikTok) and Bilibili. All searches were conducted using newly created accounts in the incognito mode of Google Chrome to avoid the influence of personalized algorithms. On each platform, the default sorting algorithm (‘Comprehensive Ranking’ on Douyin and ‘Most Relevant’ on Bilibili) was used to simulate the experience of a typical user. The top 100 relevant videos of each search term from each platform were retrieved for further screening. Data collection was carried out on 10 June 2026, to maintain consistency.

### Inclusion and exclusion criteria

Videos were included if their primary content was directly related to passive smoking. Exclusion criteria were: 1) duplicate videos; 2) irrelevant content; 3) videos with promotional content; and 4) videos <30 seconds as they typically lack sufficient content to convey health information^15^. For duplicate videos, the earlier one was retained.

### Data extraction

After applying these criteria, remaining videos were retrieved into a standardized Microsoft Excel spreadsheet: platform (Bilibili/Douyin), date of upload, video duration (seconds), and quantitative engagement metrics including the number of likes, comments, collections, and shares. Uploaders were classified into three groups based on their primary stated professional identity as indicated in their profile descriptions: ‘Doctors’ included healthcare providers (physicians and nurses); ‘Science Communicators (SCs)’ included individuals or institutions whose primary stated role was health education or science communication without a clinical practice background. ‘Lay Users (LUs)’ included individual uploaders without professional health or science credentials. The screening process was conducted by author WZ.

### Video quality assessment

The quality of the included videos was assessed using four validated tools. The Global Quality Scale (GQS) was used to evaluate the overall quality of the video^16^. It assigns a score from 1 (poor quality) to 5 (excellent quality). Detailed description is provided in Supplementary file Table 2. Reliability was measured via the modified Decision-making Information Support Criteria for Evaluating the Reliability of Non-randomized Studies (mDISCERN)^9^. It covers five items: 1) clarity of aims, 2) use of reliable sources, 3) balance of information, 4) additional information, and 5) area of uncertainty (detailed criteria are provided in Supplementary file Table 3). Each item is scored zero or one. The Journal of the American Medical Association (JAMA) benchmark criteria were used to assess video transparency^17^. Four domains, authorship, attribution, disclosure, and currency were assessed, with each domain being scored 0 or 1 (Supplementary file Table 4)^17^. The Video Information and Quality Index (VIQI) contains four sections: information flow, clarity, video quality, and consistency^18^. Each section is rated between zero and five, with the total score up to 20^18^. All videos were independently rated by two reviewers, both of whom are healthcare professionals with relevant working experience. Any disagreement in scores was resolved by a third reviewer. The inter-rater reliability was calculated using Cohen’s κ coefficient, with κ >0.60 suggesting good agreement between reviewers (Supplementary file Table 5)^19^.

### Ethical considerations

This study did not involve human or animal research. All data were extracted from publicly available content on Bilibili and Douyin, and no interactions with users were involved. Ethical approval was not required for this type of study.

### Statistical analysis

Descriptive statistics were used to demonstrate video characteristics and quality metrics. Medians and interquartile ranges (IQRs) are reported given the nature of non-parametric data distribution. The Mann-Whitney U test was used to compare non-normal distribution continuous variables among two groups, whereas the Kruskal-Wallis test was employed to assess differences in GQS, mDISCERN, JAMA benchmark criteria, and VIQI across different uploader groups. The association between nonnormally distributed continuous variables was assessed using Spearman correlation analysis. A p<0.05 was considered statistically significant. All statistical computations were conducted using R software version 4.3.3.

## RESULTS

### Video basic characteristics

**Figure 1:**
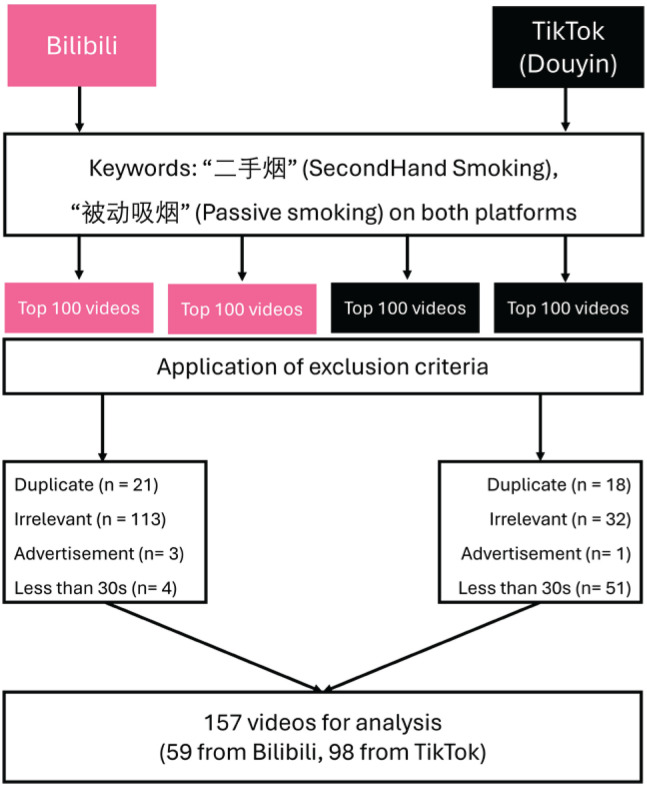
Search strategy and video selection process

In all, 400 videos were initially screened. Following exclusion criteria, 157 videos were included in the final analysis: 59 from Bilibili and 98 videos from Douyin. The detailed video selection process is illustrated in Figure 1. The basic characteristics of the included videos are presented in Table 1. Remarkable differences were observed between the two platforms. Videos on Bilibili were substantially longer than those on Douyin (median duration: 130.0 vs 73.0 s, Z=-3.93, p<0.001). In contrast, Douyin videos generated significantly greater user engagement, particularly in terms of comments (median: 89.0 vs 27.0, Z= -2.48, p<0.05) and shares (median: 1829.5 vs 72.0, Z= -4.88, p<0.001). No significant differences were identified for likes or collections.

**Table 1:** Basic information of included videos on Bilibili and Douyin from a cross‑sectional study conducted in China, June 2026 (N=157)

*Variables*	*Bilibili (N=59)* *Median (IQR)*	*Douyin (N=98)* *Median (IQR)*	*Effect size* *Z*	*p*
Duration (s)	130.00 (85.50–279.50)	73.00 (43.50–140.75)	-3.93	<0.001
Likes	398.00 (50.00–5675.00)	1113.50 (86.50–13860.75)	-1.27	0.203
Comments	27.00 (1.00–246.00)	89.00 (5.00–1196.75)	-2.48	0.013
Collections	156.00 (18.50–1407.50)	234.50 (27.00–2261.00)	-1.06	0.288
Shares	72.00 (11.00–882.50)	1829.50 (106.50–28480.75)	-4.88	<0.001
GQS	3.00 (3.00–3.00)	3.00 (3.00–4.00)	-0.83	0.406
mDISCERN	3.00 (2.00–3.00)	3.00 (2.00–3.00)	-0.35	0.725
JAMA	2.00 (2.00–3.00)	2.00 (2.00–3.00)	-0.84	0.403
VIQI	11.00 (8.00–12.00)	12.00 (11.00–15.00)	-3.37	<0.001
	***n (%)***	***n (%)***		
**Uploader**			χ²=1.17	0.557
Doctors	12 (20.34)	26 (26.53)		
LUs	21 (35.59)	28 (28.57)		
SCs	26 (44.07)	44 (44.90)		

IQR: interquartile range. Z: Mann-Whitney test. GQS: Global Quality Scale. mDISCERN: modified Decision-making Information Support Criteria for Evaluating the Reliability of Non-randomized Studies. JAMA: Journal of the American Medical Association. VIQI: Video Information and Quality Index. LUs: lay users. SCs: science communicators.

Regarding uploader types, both platforms shared a similar pattern (χ^2^=1.17, p=0.557), with science communicators (SCs) constituting the largest proportion: 44.1% on Bilibili vs 44.9% on Douyin. More institutional accounts were on Bilibli, whereas Douyin attracted more healthcare professionals (26.53% vs 20.34%).

### Video quality and reliability across platforms

**Figure 2:**
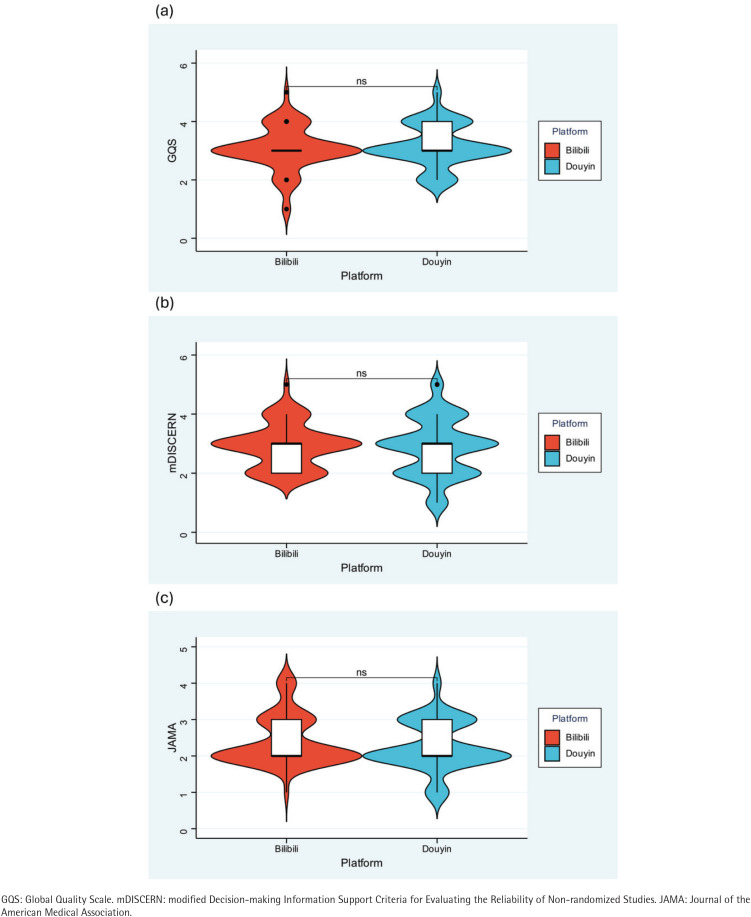
Violin plots for quality and distribution of passive smoking‑related videos across both platforms: a) GQS, b) mDISCERN, c) JAMA. The box boundaries indicate the interquartile range (IQR), while the central horizontal line represents the median value. Whiskers extend to the most extreme observations within 1.5 times the IQR from the box edges, and individual points beyond this range are displayed as outliers

The distributions of quality assessment scores are further visualized in Figure 2. Overall, passive smoking-related videos demonstrated moderate and comparable informational quality and reliability. The median GQS score was 3.0 on both platforms, indicating moderate educational value. Similarly, no significant differences were observed in mDISCERN scores (median: 3.0 for both platforms, Z= -0.35, p=0.725) or JAMA benchmark criteria (median: 2.0 for both platforms, Z= -0.84, p=0.403). These findings revealed comparable levels of reliability and transparency between Bilibili and Douyin. In contrast, significant differences showed in VIQI scores: Douyin videos achieved higher VIQI scores than Bilibili videos (median: 12.0, IQR: 11.00–15.00 vs 11.0, IQR: 8.00–12.00, Z= -3.37, p<0.001). Furthermore, the interrater reliability between the two reviewers was good across all assessment instruments: GQS (κ=0.675), mDISCERN (κ=0.643), JAMA (κ=0.745), and VIQI (κ=0.623).

### Video quality and reliability across uploader types

**Figure 3:**
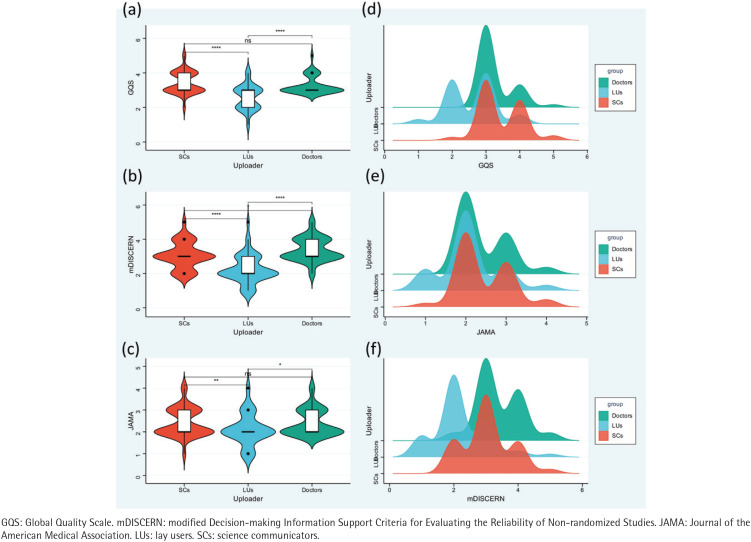
The violin and ridge plots of quality metrics distribution of videos related to passive smoking across sources: a/d) GQS, b/e) mDISCERN, c/f) JAMA. The box boundaries indicate the interquartile range (IQR), while the central horizontal line represents the median value. Whiskers extend to the most extreme observations within 1.5 times the IQR from the box edges, and individual points beyond this range are displayed as outliers

Further comparisons based on uploader category are shown in Table 2 and Figure 3. Video duration also varied significantly among uploader categories (p<0.05). Lay users uploaded the longest videos (median: 121.0 s, IQR: 79.00–282.00), whereas healthcare professionals uploaded the shortest videos (median: 71.0 s, IQR: 44.00–123.50). Social engagement metrics did not significantly differ among uploader groups. Although lay-user videos tended to accumulate higher median numbers of likes, comments, and collections, these differences did not reach statistical significance. However, significant differences were observed across different sources. Science communicators achieved the highest overall quality scores, with a median GQS of 3.0 (IQR: 3.0–4.0) and a median VIQI of 14.0 (IQR: 11.0–16.0). Doctors also produced relatively high-quality content, whereas lay users consistently obtained the lowest scores across all assessment tools. Significant differences were observed for GQS (p<0.001), mDISCERN (p<0.001), JAMA (p<0.05), and VIQI (p<0.001).

**Table 2:** Social and quality metrics of passive smoking‑related videos across uploaders from a cross‑sectional study conducted in China, June 2026 (N=157)

*Variables*	*Doctors (N=38)* *Median (IQR)*	*LUs (N=49)* *Median (IQR)*	*SCs (N=70)* *Median (IQR)*	Effect size* *χ2*	*p*
Duration (s)	71.00 (44.00–123.50)	121.00 (79.00–282.00)	98.00 (47.50–157.75)	9.79	0.007
Likes	1085.50 (84.25–4251.75)	1813.00 (159.00–45000.00)	370.00 (48.75–6035.75)	4.17	0.124
Comments	89.00 (3.00–401.00)	223.00 (7.00–3189.00)	26.00 (3.00–341.75)	3.90	0.143
Collections	185.00 (20.00–1044.25)	380.00 (53.00–6853.00)	132.50 (23.00–1636.50)	2.32	0.313
Shares	927.00 (25.50–8219.50)	284.00 (50.00–3250.00)	281.00 (33.75–7722.50)	0.06	0.971
GQS	3.00 (3.00–3.00)	3.00 (2.00–3.00)	3.00 (3.00–4.00)	39.84	<0.001
mDISCERN	3.00 (3.00–4.00)	2.00 (2.00–3.00)	3.00 (3.00–3.00)	43.75	<0.001
JAMA	2.00 (2.00–3.00)	2.00 (2.00–2.00)	2.00 (2.00–3.00)	8.02	0.018
VIQI	12.00 (11.00–13.00)	9.00 (7.00–11.00)	14.00 (11.00–16.00)	51.23	<0.001

IQR: interquartile range.

* Kruskal-Wallis test. GQS: Global Quality Scale. mDISCERN: modified Decision-making Information Support Criteria for Evaluating the Reliability of Nonrandomized Studies. JAMA: Journal of the American Medical Association. VIQI: Video Information and Quality Index. LUs: lay users. SCs: science communicators.

### Correlation analysis

**Figure 4:**
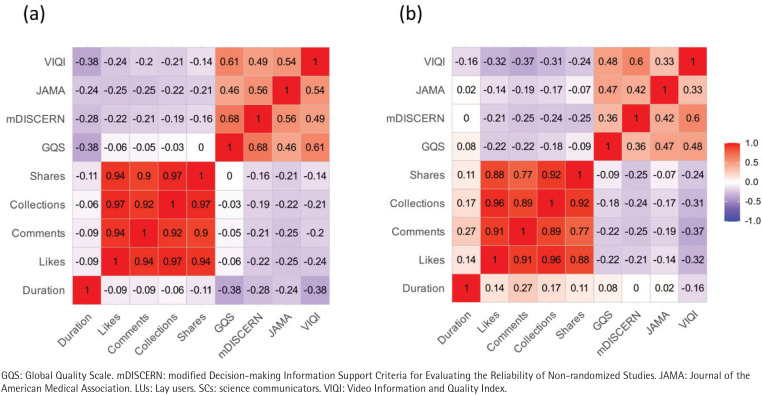
Correlation matrix of video engagement metrics and quality scores on: a) Bilibili and b) Douyin

Spearman correlation heatmaps were generated to examine the relationships between engagement metrics (duration, likes, comments, collections, shares) and quality indicators (GQS, mDISCERN, JAMA, and VIQI) for videos on both platforms (Figure 4; and Supplementary file Table 6). On Bilibili, strong positive associations were observed among engagement metrics, including likes, comments, collections, and shares (r=0.90–0.97; p<0.001 for all). The quality indicators were moderately associated with each other, with the strongest associations observed between GQS and mDISCERN (r=0.68; p<0.001) and between GQS and VIQI (r=0.61; p<0.001). In contrast, video duration showed weak negative associations with all quality measures (r= -0.24 to -0.38; p>0.05 for all). Engagement metrics were generally weakly negatively associated with quality scores (r= -0.25 to -0.03; Supplementary file Table 6). Similar patterns were observed on Douyin, engagement metrics were highly associated with one another (r=0.77–0.96; p<0.001 for all). Moderate positive associations were observed among quality assessment tools (r=0.47–0.60; p<0.001 for all). Video duration demonstrated little association with quality measures, showing near-zero associations with quality metrics (r= -0.16 to 0.08; p<0.05). Likewise, engagement metrics exhibited weak negative associations with quality scores (r= -0.37 to -0.07; Supplementary file Table 7).

## DISCUSSION

This cross-sectional study identified several meaningful observations. First, the overall quality and reliability of videos on both platforms were moderate. Second, video source was significantly associated with content quality, with videos produced by science communicators and healthcare professionals consistently demonstrating higher quality than those uploaded by lay users. Finally, audience engagement metrics were poorly associated with information quality, suggesting that popularity should not be considered a proxy for reliability.

Passive smoking remains an important public health issue worldwide. Despite strong evidence linking secondhand smoke exposure to cardiovascular disease, respiratory disorders, cancer, and adverse pregnancy outcomes, public awareness remains limited^1-3^. As social media has already become a major source of health information, particularly among younger populations, the quality of information disseminated through these platforms may influence public understanding and health-related decision-making^20^. Our findings indicate that the current passive smoking-related content available on Chinese social media platforms provides a moderate level of educational value but still leaves considerable room for improvement.

The moderate video quality observed in this study is consistent with findings from previous investigations evaluating health information on social media^12,21,22^. Cai et al.^12^ reported that videos related to gestational diabetes mellitus (GDM) on major Chinese social media platforms were generally of poor quality, despite being predominantly uploaded by medical professionals^12^. Cao et al.^21^ also identified that cataract-related videos were generally of suboptimal quality on Douyin, with content quality varying according to uploader characteristics and video content, while audience engagement metrics such as likes, comments, shares, and favorites were not associated with information quality. Similar phenomena were observed on sarcopenia-related videos on Bilibili and Douyin^22^.

These findings suggest that the patterns observed in passive smoking-related videos reflect a broader challenge within social media-based health communication. However, passive smoking may also be influenced by topic-specific factors. Tobacco control remains politically and economically sensitive in China, where the state-owned tobacco industry contributes substantially to government revenues^23^. This context may encourage self-censorship among creators and increase susceptibility to misinformation from industry-affiliated sources. These factors collectively may uniquely contribute to the moderate quality of passive smoking-related videos on social media.

Another notable finding was that Douyin videos achieved significantly higher VIQI scores despite demonstrating similar GQS, mDISCERN, and JAMA scores compared with Bilibili videos. This may be attributed to Douyin’s platform characteristics and recommendation algorithms, which encourage creators to produce visually appealing, concise, and engaging videos^22,24,25^. As a result, information may be delivered more effectively without necessarily improving scientific accuracy or reliability. Future health education initiatives may benefit from combining the scientific rigor often associated with professional content creators with the strong audiovisual production techniques commonly observed on Douyin.

Furthermore, the video source was observed to be among the strongest determinants of informational quality in our analysis. Videos uploaded by doctors and science communicators consistently achieved higher scores than those of lay persons across all quality assessment instruments. Although many videos created by lay users may be well-intentioned and based on personal experiences, such content frequently lacks supporting evidence and may oversimplify complex health issues. These findings support previous studies demonstrating that professional involvement is associated with higher quality medical information on social media platforms inside and outside China^25-27^.

Interestingly, science communicators achieved even higher VIQI scores than healthcare professionals. This observation is inconsistent with some existing studies^24,27^. Healthcare professionals often focus on clinical details, and they are usually self-reviewed, whereas science communicators may have a team to work for them. Their contents are often under more careful supervision. This does not necessarily indicate superiority of content quality, as both groups produced content of comparable GQS and mDISCERN scores, but rather suggests differences in presentation approaches.

The lack of association between engagement and quality metric also deserves attention. This result suggests that highly popular videos are not necessarily the most reliable or informative. Social media engagement is determined by numerous factors beyond educational value, including emotional appeal, entertainment value, video production quality, and algorithmic promotion^28^. Similar findings have been reported across multiple medical specialties^24,25,27^. The mixed associations between video duration and quality scores imply that longer videos may provide greater opportunities for comprehensive discussion of health topics, but they do not guarantee informational quality.

### Strengths and limitations

This study has several strengths. First, it explored passive smoking-related video content across China’s two most influential video-sharing platforms, Bilibili and Douyin, to offer valuable insights into the digital environment. Second, we used rigorous methodological procedures, including GQS, mDISCERN, JAMA benchmark criteria, and VIQI to allow a more comprehensive assessment of content quality and reliability. Moreover, dual reviewers independently rated each video, along with the calculation of inter-rater reliability. Cohen’s coefficient demonstrated good agreement. In addition, two search keywords were used to maximize the inclusion of all relevant videos.

However, several limitations should be acknowledged. Firstly, the cross-sectional design provides only a snapshot of available content and may not fully capture the rapidly evolving nature of social media platforms; additionally, it does not allow us to establish causal relationships between platform characteristics and content quality. Secondly, although focusing on Chinese social media is highly relevant given the widespread use of social media and the substantial burden of passive smoking in China, only two platforms (Bilibili and Douyin) were included, and the screening was limited to the top 100 videos. Consequently, our findings may not be fully generalizable to other Chinese platforms such as RedNotes, Kwai, or Weibo, nor to platforms in other cultural contexts, and may not capture the full spectrum of content. Future studies should incorporate broader platform coverage and more comprehensive sampling strategies. Lastly, despite the use of multiple validated assessment instruments, some degree of subjective judgment in video evaluation is unavoidable. Future research should incorporate longitudinal designs, expand analyses to additional social media platforms, and explore interventions aimed at improving the quality and reliability of health information disseminated through social media.

## CONCLUSIONS

Passive smoking-related videos on Douyin and Bilibili showed moderate quality and reliability. Douyin videos had better presentation quality and higher engagement, while the overall quality was comparable across the two platforms. Content from science communicators had the highest quality than that from doctors and lay users. Video popularity may not reflect information quality, thereby, greater professional participation and improved digital health literacy are warranted.

## Supplementary Material



## Data Availability

The data supporting this research are available from the authors on reasonable request.
