# Supplementary material for: The quality and reliability of short videos about passive smoking on Bilibili and TikTok: A cross-sectional study
Source: Tob Induc Dis. 2026 Aug 5;24:10.18332/tid/225461. doi: 10.18332/tid/225461 (PMC13450130; doi:10.18332/tid/225461)
Supplement: Supplementary file 1 [file TID-24-140-s1.pdf]

Tobacco Induced Diseases

Supplementary file

© 2026 Zhang W. et al.

DOI:
10.18332/tid/225461

The content has been provided by the author(s) and has not been reviewed, verified, or endorsed by European Publishing. It may not have undergone peer review. The views, opinions, and recommendations expressed are solely those of the author(s) and do not necessarily reflect the position of European Publishing. European Publishing accepts no responsibility or liability for any consequences arising from the use of, or reliance on, this content.

Supplementary Table 1. STROBE statement: checklist of items that should be included in reports of cross-sectional studies

	Item No	Recommendation	Page No
**Title and abstract**	1	(*a*) Indicate the study's design with a commonly used term in the title or the abstract	1
		(*b*) Provide in the abstract an informative and balanced summary of what was done and what was found	1-2
**Introduction**			
Background/rationale	2	Explain the scientific background and rationale for the investigation being reported	3-4
Objectives	3	State specific objectives, including any prespecified hypotheses	3-4
**Methods**			
Study design	4	Present key elements of study design early in the paper	4-5
Setting	5	Describe the setting, locations, and relevant dates, including periods of recruitment, exposure, follow-up, and data collection	4-5
Participants	6	(*a*) Give the eligibility criteria, and the sources and methods of selection of participants	4-7

Variables	7	Clearly define all outcomes, exposures, predictors, potential confounders, and effect modifiers. Give diagnostic criteria, if applicable	4-7
Data sources/ measurement	8*	For each variable of interest, give sources of data and details of methods of assessment (measurement). Describe comparability of assessment methods if there is more than one group	4-7
Bias	9	Describe any efforts to address potential sources of bias	4-7
Study size	10	Explain how the study size was arrived at	4-7
Quantitative variables	11	Explain how quantitative variables were handled in the analyses. If applicable, describe which groupings were chosen and why	4-7
Statistical methods	12	(*a*) Describe all statistical methods, including those used to control for confounding	7
		(*b*) Describe any methods used to examine subgroups and interactions	7
		(*c*) Explain how missing data were addressed	7
		(*d*) If applicable, describe analytical methods taking account of sampling strategy	7
		(*e*) Describe any sensitivity analyses	N/A

Results			
Participants	13*	(a) Report numbers of individuals at each stage of study-eg numbers potentially eligible, examined for eligibility, confirmed eligible, included in the study, completing follow-up, and analysed	7-9
		(b) Give reasons for non-participation at each stage	Fig1.
		(c) Consider use of a flow diagram	Fig1.
Descriptive data	14*	(a) Give characteristics of study participants (eg demographic, clinical, social) and information on exposures and potential confounders	Table 1
		(b) Indicate number of participants with missing data for each variable of interest	N/A
Outcome data	15*	Report numbers of outcome events or summary measures	7-10
Main results	16	(*a*) Give unadjusted estimates and, if applicable, confounder-adjusted estimates and their precision (eg, 95% confidence interval). Make clear which confounders were adjusted for and why they were included	7-10
		(*b*) Report category boundaries when	7-10

		continuous variables were categorized	
		(*c*) If relevant, consider translating estimates of relative risk into absolute risk for a meaningful time period	7-10
Other analyses	17	Report other analyses done-eg analyses of subgroups and interactions, and sensitivity analyses	7-10
**Discussion**			
Key results	18	Summarise key results with reference to study objectives	10-13
Limitations	19	Discuss limitations of the study, taking into account sources of potential bias or imprecision. Discuss both direction and magnitude of any potential bias	14-15
Interpretation	20	Give a cautious overall interpretation of results considering objectives, limitations, multiplicity of analyses, results from similar studies, and other relevant evidence	11-13
Generalisability	21	Discuss the generalisability (external validity) of the study results	11-13
**Other information**			
Funding	22	Give the source of funding and the role of the funders for the present study and, if	NA

	applicable, for the original study on which the present article is based	

*Give information separately for exposed and unexposed groups. STROBE:

Note: An Explanation and Elaboration article discusses each checklist item and gives methodological background and published examples of transparent reporting. The STROBE checklist is best used in conjunction with this article (freely available on the Web sites of PLoS Medicine at http://www.plosmedicine.org/, Annals of Internal Medicine at http://www.annals.org/, and Epidemiology at http://www.epidem.com/). Information on the STROBE Initiative is available at www.strobe-statement.org.

Supplementary Table 2. GQS quality criteria

Item features	Points
Poor quality; poor flow of the videos; most information missing; not at all useful for patients	1
Generally poor quality; some information listed, but many important topics missing; of very limited use to patients	2
Moderate quality; suboptimal flow; some important adequately discussed, but other information poorly discussed; somewhat useful for patients	3
Good quality and generally good flow; most of the relevant information listed, but some topics not covered; useful for patients	4
Excellent quality and flow; very useful for patients	5

GQS: Global Quality Score

Supplementary Table 3. The mDISCERN quality criteria

Reliability Score	
1. Is the video clear, concise, and understandable?	Yes (1) / No (0)
2. Are valid sources cited?	Yes (1) / No (0)
3. Is the content presented balanced and unbiased?	Yes (1) / No (0)
4. Are additional sources of content listed for patient reference?	Yes (1) / No (0)
5. Are areas of uncertainty mentioned?	Yes (1) / No (0)

mDISCERN: Modified Decision-making Information Support Criteria for Evaluating the Reliability of Non-randomized Studies

Supplementary Table 4. The JAMA benchmark criteria

**Criteria (1 point for each item)**	**Description**
**Authorship**	Author and contributor credentials and their affiliations should be provided
**Attribution**	Clearly lists all copyright information and states references and sources for content
**Currency**	Initial date of posted content and subsequent updates to content should be provided
**Disclosure**	Conflicts of interest, funding, sponsorship, advertising, support, and video ownership should be fully disclosed

JAMA: Journal of the American Medical Association

Supplementary Table 5. The inter-rater reliability of quality metrics

	GQS	mDISCERN	JAMA	VIQI
**Cohen's κ coefficients**	0.675	0.643	0.745	0.623

GQS: Global Quality Scale, mDISCERN: Modified Decision-making Information Support Criteria for Evaluating the Reliability of Non-randomized Studies, JAMA: Journal of the American Medical Association, VIQI: Video Information and Quality Index.

Supplementary Table 6. The p-value for Spearman correlation analysis for videos from Bilibili

Variable	Duration	Likes	Comments	Collections	Shares	GQS	mDISCERN	JAMA	VIQI
**Duration**	-	0.282	0.041	0.212	0.425	0.529	0.973	0.888	0.227
**Likes**		-	< 0.001	< 0.001	< 0.001	0.088	0.108	0.281	0.013
**Comments**			-	< 0.001	< 0.001	0.087	0.053	0.153	0.004
**Collections**				-	< 0.001	0.182	0.066	0.200	0.019
**Shares**					-	0.485	0.053	0.610	0.066
**GQS**						-	< 0.001	< 0.001	< 0.001
**mDISCERN**							-	< 0.001	< 0.001
**JAMA**								-	0.010
**VIQI**									-

GQS: Global Quality Scale, mDISCERN: Modified Decision-making Information Support Criteria for Evaluating the Reliability of Non-randomized Studies, JAMA: Journal of the American Medical Association, VIQI: Video Information and Quality Index.

Supplementary Table 7. The p-value for Spearman correlation analysis for videos from Douyin (TikTok)

Variable	Duration	Likes	Comments	Collections	Shares	GQS	mDISCERN	JAMA	VIQI
**Duration**	-	0.390	0.380	0.550	0.296	< 0.001	0.005	0.019	< 0.001
**Likes**		-	< 0.001	< 0.001	< 0.001	0.554	0.028	0.013	0.018
**Comments**			-	< 0.001	< 0.001	0.633	0.037	0.014	0.054
**Collections**				-	< 0.001	0.507	0.067	0.030	0.038
**Shares**					-	0.327	0.107	0.035	0.163
**GQS**						-	< 0.001	< 0.001	< 0.001
**mDISCERN**							-	< 0.001	< 0.001
**JAMA**								-	< 0.001
**VIQI**									-

GQS: Global Quality Scale, mDISCERN: Modified Decision-making Information Support Criteria for Evaluating the Reliability of Non-randomized Studies, JAMA: Journal of the American Medical Association, VIQI: Video Information and Quality Index.
